# PICO Portal

**DOI:** 10.29173/jchla29590

**Published:** 2021-12-01

**Authors:** Joel T Minion, Oluwaseun Egunsola, Liza Mastikhina, Brenlea Farkas, Mark Hofmeister, Jordyn Flanagan, Charleen Salmon, Fiona Clement

**Affiliations:** Health Technology Assessment Unit, O’Brien Institute for Public Health, Cumming School of Medicine, University of Calgary, Calgary, Alberta, Canada

## Abstract

PICO Portal is a Web-based systematic review management tool launched in September 2020 to better facilitate collaborative knowledge synthesis in biomedical research. Most notably, it uses machine learning and Natural Language Processing algorithms to continuously refine the screening process by analyzing decisions as made by the review team. PICO Portal was evaluated by researchers with the Health Assessment Technology team at the University of Calgary, who routinely undertake PICO-based systematic reviews, currently using an in-house manual system. The team appreciated many aspects of PICO Portal and felt it held considerable promise to better support the review process. At the same time, they found it wasn’t as user-friendly as expected and would benefit from additional refinement if it is to appeal to a wider range of users, particularly those less familiar with the systematic review process.

**Product:** PICO Portal

**URL:** http://www.picoportal.org

## Product Description

PICO Portal is a Web-based tool that allows research teams to work collaboratively on PICO-based (Population, Intervention, Comparison, Outcomes) systematic or scoping reviews. It uses artificial intelligence (AI) to learn from decisions made as records are screened and to highlight keywords in relation to each element of the PICO. AI also continuously assesses and sorts citations, moving those most likely to meet the inclusion criteria to the front of queue, concentrating team efforts on papers of greatest probable relevance. Such filtering also allows staffing requirements to be ‘front loaded’ to match the higher volume of screening work characteristic of the earlier stages of a systematic review.

At its heart, the tool facilitates and manages standard elements of the review process: uploading search results from multiple bibliographic databases to a central location; de-duplicating records and helping source PDFs; customizing inclusion/exclusion criteria; assigning and overseeing screening efforts; and tagging and extracting data for analysis. All activity is monitored and recorded through a central dashboard, which in turn provides analytics by which to scrutinize the review.

## Purpose and Intended Audience

PICO Portal is designed to enhance and speed up the systematic review process. It is targeted specifically to PICO-based reviews and is best suited for more experienced academic research teams, including those working remotely or from multiple geographic locations. Given its focus and complexity, the tool is less suitable for simpler reviews, researchers less familiar with the systematic review process, and reviews that are not PICO-based.

## Cost

Currently free, although the licensing agreement allows for the introduction of tiered subscription charges based on an account holder’s organizational status and number of projects.

## Special Features

There are a number of features that set PICO Portal apart from our current manual approach. These include:

- Use of machine learning and natural language processing algorithms to facilitate the screening process.
- Ready access to the PICO criteria along with colour coding of keywords to each PICO element.
- An information-rich central dashboard to gauge and manage the review process.
- A capacity to see who else is working on the project as well as their role and activity.
- Analytics to help calibrate screener accuracy and monitor decisions.
- Easy standardization and refinement of reasons for exclusion in relation to the PICO.
- Integration of PDFs (if uploaded) into the review interface alongside abstracts.
- An ability to download updated metrics and outputs as Excel spreadsheets (e.g. keyword frequency, user analytics, PRISMA flowchart).

## Usability

Overall, PICO Portal does what it should: it helps researchers and librarians upload and screen large sets of search results to extract pertinent data for analysis. Where it stumbles is in its interface. The tool is not always intuitive to use and can be downright frustrating. For example, our team struggled with basic tasks like uploading search results and inviting new team members without needing to refer to the WIKI resources, which are themselves rather limited. Links are not always where expected and labels are sometimes less than self-explanatory. PICO Portal also presumes a relatively high degree of familiarity with the PICO-based systematic review process to be used effectively. Early career researchers and students may struggle without support from a more experienced biomedical librarian or researcher. They should allow sufficient time to learn the tool before using it to undertake a review.

## Compatibility Issues

The tool purports to be supported by the most common browsers (Chrome, Firefox, Edge, Safari). Our team reported no compatibility issues.

## Comparison with Similar Products

PICO Portal sits alongside several other systematic review management tools such as DistillerSR, Covidence, EPPI-Reviewer, and SWIFT Active Screener. Members of our team familiar with the first two judged PICO Portal as being broadly on par in terms of overall usability. Given that these other tools are generally fee-based, PICO Portal has the central advantage of being free.

## Strengths

- Uses AI to help innovate management of the review process.
- Excellent main dashboard for monitoring activity and exporting results as Excel documents.
- Available at no cost to individual and academic users.
- Within certain limitations, a viable choice for PICO-based systematic review management.

## Weaknesses

- Not always intuitive to use, with at best adequate support resources (videos, FAQs).
- Unless uploaded initially alongside search results, PDFs need to be pulled manually.
- Inability to see inclusion/exclusion criteria when screening individual records.
- Less than refined (though still workable) data extraction process.
- Likely challenging for less experienced researchers or students to master.
- No indication of where project data are stored or what systems are in place to ensure data security or back-up.

## Conclusion

PICO Portal is a fully functioning and free systematic review management tool that demonstrates the potential of AI to enhance the review process. In its current form it would be particularly useful for health librarians and biomedical research teams already conversant with PICO-based reviews. In practice, PICO Portal is let down in a few areas, including not being overly intuitive to use, requiring a steep learning curve for some users, and a lack of transparency in where and how projects are stored. Like all such management tools, PICO Portal faces the challenge of needing to be such an improvement on current practice that it can entice researchers away from systems they already know. In this respect, while our team will keep an eye on PICO Portal given its innovative potential, we have decided to stay with our more manual in-house system for now.

